# Simple severity scale for perforated peptic ulcer with generalized peritonitis: a derivation and internal validation study

**DOI:** 10.1097/JS9.0000000000002037

**Published:** 2024-08-08

**Authors:** Ryo Yamamoto, Shinya Hirakawa, Hisateru Tachimori, Tadashi Matsuoka, Hirotoshi Kikuchi, Hiroshi Hasegawa, Ken Shirabe, Yoshihiro Kakeji, Hirofumi Kawakubo, Yuko Kitagawa, Junichi Sasaki

**Affiliations:** a Department of Emergency and Critical Care Medicine, Keio University School of Medicine, 35 Shinanomachi, Shinjuku; b Endowed Course for Health System Innovation, Keio University School of Medicine; c Department of Healthcare Quality Assessment, Graduate School of Medicine, The University of Tokyo; d Project Management Subcommittee, The Japanese Society of Gastroenterological Surgery, Axior Mita 6F, Mita, Minato-ku; e The Japanese Society of Gastroenterological Surgery, Axior Mita 6F, Mita, Minato-ku; f Database Committee, The Japanese Society of Gastroenterological Surgery, Axior Mita 6F, Mita, Minato-ku; g Department of Surgery, Keio University School of Medicine, 35 Shinanomachi, Shinjuku, Tokyo, Japan

**Keywords:** emergency laparotomy, gastrointestinal perforation, and prediction model, peptic ulcer disease

## Abstract

**Background:**

Perforated peptic ulcer (PPU) causes peritonitis and requires surgery based on disease severity. This study aimed to develop and validate a severity scale for PPU with generalized peritonitis.

**Materials and methods:**

This retrospective cohort study used a nationwide multicenter surgical database (2013–2020). Patients aged >15 years who underwent surgery for PPU with generalized peritonitis were included and categorized into the derivation (2013–2018) and two validation (2019 and 2020) cohorts. Possible severity predictors were selected via a literature review, and Lasso models were developed to predict severe postoperative adverse events with 2000 bootstrapping. Final variables for the scoring system were determined based on inclusion frequency (≥90%) in the Lasso models. Discrimination and accuracy were evaluated using C-statistics and calibration plots. Cutoff values for minimal postoperative adverse events were examined using negative predictive values.

**Results:**

Among 12 513 patients included (1202 underwent laparoscopic surgery), 533 (5.9%), 138 (7.6%), and 117 (6.9%) in the derivation and two validation cohorts experienced postoperative adverse events. Age, dyspnea at rest, preoperative sepsis, III/IV/V of American Society of Anesthesiologists physical status, and albumin and creatinine were selected for the final model. A 0–11 scoring system was developed with C-statistics of 0.812–0.819. Cutoff value was determined as 5, which predicted <3% probability of postoperative adverse events regardless of type of surgery.

**Conclusions:**

A score of <5 predicts minimal risks for postoperative adverse events and, therefore, would be clinically useful to determine the type of surgery. Further studies are needed to validate the score.

## Introduction

Highlights

- Simple 0–11 scoring system for perforated peptic ulcer with peritonitis was developed by Lasso models using national database.
- Score included only preoperative information (age, respiratory distress, sepsis, American Society of Anesthesiologists physical status, albumin, and creatinine).
- Score ≥5 had 97.5–97.9% of negative predictive value for postoperative adverse events.

Peptic ulcer disease is a major gastrointestinal illness commonly caused by *Helicobacter pylori* infection and the chronic use of nonsteroidal anti-inflammatory drugs^1,2^. Although proton pump inhibitors can be used to manage peptic ulcers and prevent disease progression, a significant number of patients still experience gastric/duodenal perforation^3,4^. Peritonitis with abdominal sepsis caused by the spillage of intragastric contents is among the most severe conditions requiring surgical intervention and introduces unfavorable outcomes, including mortality^5^.

There are several treatment options for perforated peptic ulcer (PPU), such as nonsurgical management with stomach decompression, laparoscopic repair of perforation, and extensive intra-abdominal lavage under laparotomy^6–9^. Although surgeons typically determine the treatment strategy based on the degree of intra-abdominal contamination, frailty status, comorbidities, and hemodynamic instability^10–12^, the definitive criteria for each management remain unclear^5,6,13^. In particular, while laparoscopic skills have advanced over the last decade^14,15^, there is ongoing discussion regarding the ideal candidates for less invasive surgery using laparoscopy.

Considering that the treatment effects of each surgery cannot be validated owing to heterogeneity in the preoperative characteristics of patients, patient stratification can be used to identify appropriate strategies for PPU^16–18^. A recent survey conducted by an international study group revealed that the preexisting peptic ulcer severity scale used different clinical descriptors, likely limited by data availability. Moreover, it identified 27 clinical factors as a core descriptor set to categorize patients^5^. Although the use of all suggested factors may not be clinically feasible, the development of a simple severity scale incorporating these well-accepted clinical parameters can enhance future research and assist surgeons in determining an appropriate surgical strategy.

Accordingly, this study aimed to stratify patients via the derivation and validation of a simple severity scale for PPU, to eventually elucidate the subgroups of patients who can benefit from each surgical intervention for PPU. Considering that the existence of generalized peritonitis is a definitive indication for surgery, a simple scoring system was developed using data from patients with PPU experiencing generalized peritonitis.

## Materials and methods

### Study design and setting

This was a retrospective cohort study conducted using multicenter data from the National Clinical Database (NCD) in Japan, which was established in 2010 as the primary database system for the Japanese board certification of surgery. The NCD contains patient records on >95% of surgeries performed in Japan, with more than 5000 institutions enrolled and over 10 million cases registered. Eligibility criteria for the NCD are available online (https://www.ncd.or.jp/). The NCD portal system requires all data to be filled, with the exception of laboratory data not obtained for the patient. Audits are conducted annually, and validation studies on the accuracy of the NCD data have shown high overall concordance, particularly in gastroenterological surgery^19,20^. The NCD includes all types of gastroenterological surgery, and detailed data on pre-/intra-/postoperative information are available for eight predefined surgical procedures, including laparotomy for generalized peritonitis.

All collaborating hospitals obtained individual local institutional review board approval for patient enrollment in the NCD that adhered to the tenets of the Declaration of Helsinki. The current study was approved by the Ethics Committee for Conduct of Human Research at the head institution of the study (application number: 20211020) before study initiation. The need for informed consent was waived owing to data anonymity. The study was retrospectively registered in the University Hospital Medical Information Network Clinical Trial Registry. Reporting of the current study adhered to the Strengthening the Reporting of Cohort Studies in Surgery (STROCSS) criteria^21^ (Supplemental Digital Content 1, http://links.lww.com/JS9/D286).

### Study population

Data from the NCD were retrospectively reviewed between 2013 and 2020. The inclusion criteria were as follows: (1) patients aged ≥15 years (including the youth), (2) those with PPU in the stomach or duodenum (with an ICD-10 code of K25.1, K25.2, K25.5, K25.6, K26.1, K26.2, K26.5, or K26.6, and (3) those who underwent open laparotomy or laparoscopy for generalized peritonitis. Patients with a co-diagnosis of other gastrointestinal perforation (e.g. perforated diverticulitis) and duplicate cases were excluded from the analysis. The diagnosis of PPU was determined based on intraoperative findings, and generalized peritonitis was determined when inflammation of the entire peritoneal cavity existed based on both physical examination and intraoperative findings.

### Data collection and definition

The case report forms obtained from the NCD included data on demographic characteristics; preoperative information, such as date of admission and surgery, emergency transport and surgery, preoperative chemo-/radiation-/immunotherapy, comorbidities with severity, activities of daily living, respiratory status based on symptoms, presence of sepsis and coagulopathy, transfusion, and laboratories; intraoperative information, such as surgical procedures with ICD-10 codes, types and risks of anesthesia, operative time, volume of blood loss and transfusion; and postoperative information, such as postoperative adverse events, length of hospital stay, and survival status at discharge and 30 days after surgery.

Emergency transport was defined as transportation via ambulance. Emergency surgery was defined as a procedure urgently performed within 24 h after the decision for surgery was made by the treating surgeon. The risk of anesthesia was categorized based on the American Society of Anesthesiologists physical status (ASA-PS) classification system^22^. Preoperative sepsis was diagnosed based on either sepsis-3 criteria or sepsis-2 criteria for severe sepsis (infectious disease with organ dysfunction required for sepsis diagnosis)^23,24^, depending on the year of surgery. Postoperative adverse events were categorized using the Clavien–Dindo classification system^25^.

### Outcome measures

The primary outcome was a grade IV or V postoperative severe adverse event based on the Clavien–Dindo classification system. Grade IV indicates a life-threatening complication requiring intensive care, and grade V represents mortality. The secondary outcomes included survival status at discharge and 30 days after surgery, length of hospital stay, and prolonged (>30 days) hospital stay.

### Statistical analysis

Descriptive data are presented as median (interquartile range) or number (%). All statistical analyses were conducted using R software version 4.1 and later versions (R Foundation for Statistical Computing) with the add-on package ‘mice’ version 3.14 and later versions for multiple imputations and ‘glmnet’ version 4.1-4 and later versions for Lasso.

#### Sample size evaluation

After the patients were selected based on the eligibility criteria, they were assigned to either the derivation or validation cohorts based on the date of surgery. Patients who underwent surgery between 2013 and 2018 were included in the derivation cohort, whereas those who underwent surgery in 2019 and 2020 were included in two validation cohorts, with the one in 2020 postdating the coronavirus disease 2019 (COVID-19) pandemic.

Sample sizes of the three cohorts were evaluated following standard methods for sample size estimation in the derivation and validation of the clinical prediction model: At least 10 outcomes for each potential predictor in the derivation cohort and at least 100 outcome events in each validation cohort. In this study, the derivation cohort had >500 outcome events (where <50 potential predictive variables could be assessed), and both validation cohorts had >100 outcome events^26–28^.

#### Data preparation

Before developing the scoring system, missing values in the derivation cohort were substituted with a set of plausible values by creating 10 filled-in complete datasets using the multiple imputation by chained equation method. Variables with ≥10% missing values, deemed clinically infeasible, were not imputed nor considered as potential severity predictors^29^.

To develop an accurate model to predict postoperative adverse events, potential predictive variables underwent logarithmic transformation or categorization, if needed, based on previous studies. The details of categorization were summarized in Supplementary Data S1 (Supplemental Digital Content 2, http://links.lww.com/JS9/D287)^26,30^.

#### Score derivation

A simple scoring system for the severity of PPU with generalized peritonitis, named the Simple PPUP score, was derived using the simple BLaF (bootstrap resampling with Lasso selection) model, which required following steps^31^.

First, 200 bootstrap resampling datasets were generated from each imputed dataset (2000 datasets in total, each has the same sample size of the original data). Then, potential severity predictors were selected based on previous studies found in a literature review. All variables in the core descriptor set for PPU, developed by an international study group using the Delphi method in 2022 (Table S1, Supplemental Digital Content 3, http://links.lww.com/JS9/D288), were considered as potential predictors.

Second, Lasso model to predict postoperative adverse events was developed in each bootstrap dataset using the potential severity predictors, in which confounding effects between variables were adjusted. As the Lasso model utilizes a penalty term in regression model, a balance between model simplicity and accuracy can be determined and therefore avoid overfitting^31^.

Third, among 2000 Lasso models that were generated in each dataset, potential predictors shown in ≥1800 models (>90% of ‘inclusion frequency’, the current study used strict criteria to only select highly influenced predictor) were selected for the final model.

Fourth, before the score was derived based on the coefficients in the final model, the final model was evaluated by comparing the C-statistic with preexisting prediction models, such as the ASA-PS classification system, Boey score, peptic ulcer perforation score (PULP score), and Acute Physiology and Chronic Health Evaluation II score^32^. Additionally, a confusion matrix was generated using the Youden index to evaluate prediction powers. Moreover, as an additional plan, we prepared to develop another more simplified model with fewer than six predictors using only variables with a 100% inclusion frequency in the Lasso models.

Finally, the Simple PPUP score was derived from the final model, wherein the coefficients of selected predictors were equally multiplied, rescaled, and then discretized to whole numbers.

#### Score validation

The Simple PPUP score was validated using two validation cohorts. Receiver operating characteristic (ROC) curves were drawn, and C-statistics with a 95% CI were calculated using 2000 bootstrap datasets. The practicality of the Simple PPUP score was examined by comparing the score–estimated probability of postoperative adverse events with the observed rate. Additionally, sensitivity analyses were performed to confirm the robustness of the Simple PPUP score^31,33^. The Simple PPUP score was analyzed using logistic regression and linear regression models for primary and secondary outcomes, as appropriate, with the Simple PPUP score entered as a continuous variable. Furthermore, considering that a grade III adverse event of the Clavien–Dindo classification is also a significant outcome, c-statistics of the Simple PPUP score for predicting grade III/VI/V adverse events were calculated.

A clinically feasible cutoff value was examined by calculating the sensitivity, specificity, positive predictive value, and negative predictive value (NPV). To evaluate clinical applicability, the number of patients who underwent each type of surgery were calculated with each cutoff value for the Simple PPUP score.

## Results

### Characteristics of the patients

**Figure 1:**
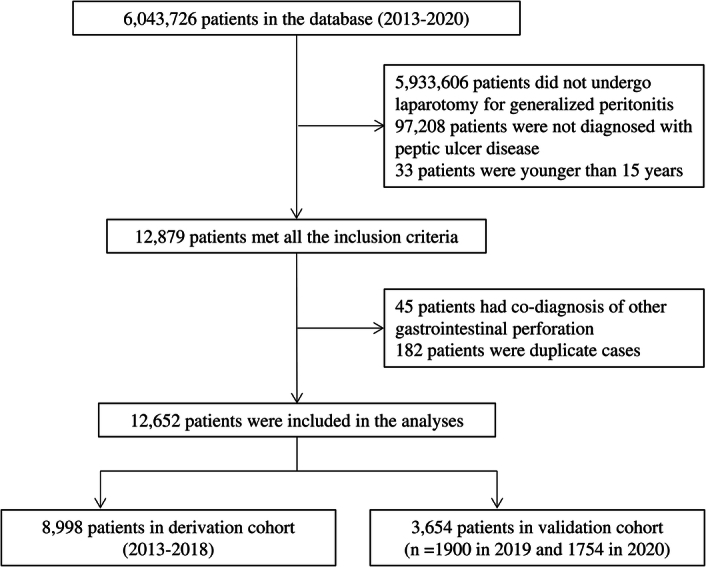
Patient flow diagram. Among 6 043 726 patients in the NCD, 12 652 were eligible for inclusion in this study. Of these patients, 8998 were assigned to the derivation cohort and 1900 and 1754 were assigned to the two validation cohorts.

Among 6 043 726 patients in the NCD, 12 652 aged ≥15 years presented with PPU and underwent laparotomy (including open and laparoscopic surgery) for generalized peritonitis. Therefore, they were eligible for inclusion in the study (Fig. 1). In total, 8998 patients were included in the derivation cohort (2013–2018) and 1900 and 1754 in the two validation cohorts (2019 and 2020, respectively).

The patient characteristics are shown in Table 1. The cohorts exhibited similar characteristics regardless of the COVID-19 pandemic. More than 50% of the patients were transported via ambulance (emergency transport), and ~95% underwent emergency surgery. Open laparotomy was conducted in ~85–90% of the included patients, wherein omental patch was the most frequent procedure. Furthermore, <5% of patients presented with comorbidities, and the validation cohorts had a relatively higher rate of anticoagulant use than the derivation cohort. Approximately 40% of patients with critical conditions underwent surgery (class III/IV/V based on the ASA-PS classification system).

**Table 1:** Characteristics of the participants.

	Derivation cohort (2013–2018)	Validation cohort (2019)	Validation cohort (2020)
Case, *n*	8998	1827	1688
Emergency transport, *n* (%)	5789 (64.3%)	1208 (66.1%)	1148 (68.0%)
Emergency surgery, *n* (%)	8627 (95.9%)	1739 (95.2%)	1601 (94.8%)
Open laparotomy, *n* (%)			
Omental patch	5263 (58.5%)	1045 (57.2%)	942 (55.8%)
Primary closure	1165 (12.9%)	297 (16.3%)	308 (18.2%)
Partial gastrectomy	247 (2.7%)	51 (2.8%)	33 (2.0%)
Total gastrectomy	24 (0.3%)	5 (0.3%)	1 (0.1%)
Laparoscopic surgery, *n* (%)			
Omental patch/primary closure	697 (7.7%)	255 (14.0%)	250 (14.8%)
Partial gastrectomy	3 (0.0%)	1 (0.1%)	0 (0.0%)
Demographic characteristics			
Age, years, median (IQR)	65 (53–78)	68 (56–78)	69 (57–80)
Male sex, *n* (%)	6186 (68.7%)	1220 (66.8%)	1105 (65.5%)
BMI, median (IQR)	21 (19–23)	21 (19–23)	21 (19–23)
Dependent ADL	2079 (23.1%)	427 (23.4%)	410 (24.3%)
Habitual drinking	2527 (28.1%)	524 (28.7%)	450 (26.7%)
Comorbidities and medications, *n* (%)			
Diabetes	1084 (12.0%)	243 (13.3%)	203 (12.0%)
COPD	224 (2.5%)	45 (2.5%)	41 (2.4%)
Congestive heart failure	124 (1.4%)	22 (1.2%)	28 (1.7%)
Myocardial infarction	25 (0.3%)	9 (0.5%)	3 (0.2%)
Angina	65 (0.7%)	9 (0.5%)	12 (0.7%)
Post-PCI	75 (0.8%)	16 (0.9%)	18 (1.1%)
Peripheral vascular disease - history of surgery	26 (0.3%)	3 (0.2%)	2 (0.1%)
Peripheral vascular disease - symptomatic	23 (0.3%)	4 (0.2%)	9 (0.5%)
Acute kidney injury	244 (2.7%)	50 (2.7%)	48 (2.8%)
Hemodialysis	131 (1.5%)	30 (1.6%)	31 (1.8%)
Cerebrovascular disease	241 (2.7%)	68 (3.7%)	68 (4.0%)
Metastatic carcinoma	156 (1.7%)	45 (2.5%)	33 (2.0%)
Preoperative chemotherapy	269 (3.0%)	78 (4.3%)	84 (5.0%)
Preoperative immunotherapy	15 (0.2%)	6 (0.3%)	3 (0.2%)
Long-term steroid use	186 (2.1%)	37 (2.0%)	43 (2.5%)
Anticoagulants	217 (2.4%)	102 (5.6%)	119 (7.0%)
Preoperative conditions, n (%)			
Respiratory distress–dyspnea on exertion	174 (1.9%)	44 (2.4%)	33 (2.0%)
Respiratory distress–dyspnea at rest	177 (2.0%)	34 (1.9%)	41 (2.4%)
Preoperative sepsis	550 (6.1%)	171 (9.4%)	167 (9.9%)
ASA-PS, class I/II	5789 (64.3%)	1088 (59.6%)	1001 (59.3%)
ASA-PS, class III/IV/V	3209 (35.7%)	739 (40.4%)	687 (40.7%)
Laboratories			
Hemoglobin level, < normal range^a^	3696 (41.1%)	837 (45.8%)	799 (47.3%)
Hemoglobin level, > normal range^a^	601 (6.7%)	126 (6.9%)	121 (7.2%)
Albumin level, 2.0–3.0 g/dl	1834 (20.4%)	447 (24.5%)	455 (27.0%)
Albumin level, <2.0 g/dl	394 (4.4%)	91 (5.0%)	90 (5.3%)
BUN level, >20 g/dl	4131 (45.9%)	879 (48.1%)	900 (53.3%)
Creatinine level, mg/dl	0.8 (0.7–1.2)	0.9 (0.7–1.3)	0.9 (0.7–1.4)
CRP level, mg/dl, >1.0	4935 (54.8%)	1054 (57.7%)	1041 (61.7%)
Outcomes			
Postoperative adverse event^b^, *n* (%)	533 (5.9%)	138 (7.6%)	117 (6.9%)
Length of hospital stay, days, median (IQR)	16 (11–28)	16 (12–28)	18 (12–31)
Prolonged hospital stay^c^, *n* (%)	1925 (21.4%)	397 (21.7%)	421 (24.9%)
30-day mortality, *n* (%)	386 (4.3%)	92 (5.0%)	89 (5.3%)
In-hospital mortality, *n* (%)	557 (6.2%)	138 (7.6%)	125 (7.4%)

^a^ Normal range of hemoglobin is 13.5–17 g/dl for male and 11.5–15 g/dl for female.

^b^ Postoperative severe adverse events defined as IV or V of Clavien–Dindo classification.

^c^ Prolonged hospital stay is defined as longer than 30 days of hospital stay.

ADL, activity of daily living; ASA-PS, American Society of Anesthesiologists physical status; BUN, blood urea nitrogen; COPD, chronic obstructive pulmonary disease; CRP, C-reactive protein; IQR, interquartile range; PCI, percutaneous coronary intervention.

Postoperative severe adverse events were observed in 533 (5.9%) patients in the derivation cohort and in 138 (7.6%) and 117 (6.9%) patients in the two validation cohorts. The mortality rates at discharge and 30 days after surgery were 6.2–7.6% and 4.3–5.3%, respectively.

### Score derivation

Age, dyspnea at rest, preoperative sepsis, class III/IV/V based on the ASA-PS classification system, and albumin and creatinine levels, were finally selected (Tables S2, Supplemental Digital Content 3, http://links.lww.com/JS9/D288 and S3, Supplemental Digital Content 3, http://links.lww.com/JS9/D288). The C-statistic of the final model was 0.863 (0.848–0.877) based on the ROC curve for predicting postoperative adverse events. This finding was similar to the C-statistic of other scaling systems (0.70–0.83) reported in previous studies with a large sample size. The sensitivity, specificity, and positive predictive value were 82, 77, and 18%, respectively, based on the Youden Index in the ROC curve. Another simplified model was not created because the final model only used six parameters.

Based on the coefficients of the six parameters in the final model, a Simple PPUP scoring system was derived (Table 2). Although the Simple PPUP score can be 0–11, the score ranged from 1 to 9 in the current study.

**Table 2:** Simple PPUP score.

Parameters	Scores
Age (years)	
15–16	0
17–50	1
51–84	2
≥85	3
Dyspnea at rest	1
Preoperative sepsis	1
ASA-PS class III/IV/V	1
Albumin level	
≥3.0	0
2.0–3.0	1
<2.0	2
Creatinine level	
≤0.5	0
0.5–1.96	1
1.96–7.56	2
>7.56	3

ASA-PS, American Society of Anesthesiologists physical status.

### Score validation

**Figure 2:**
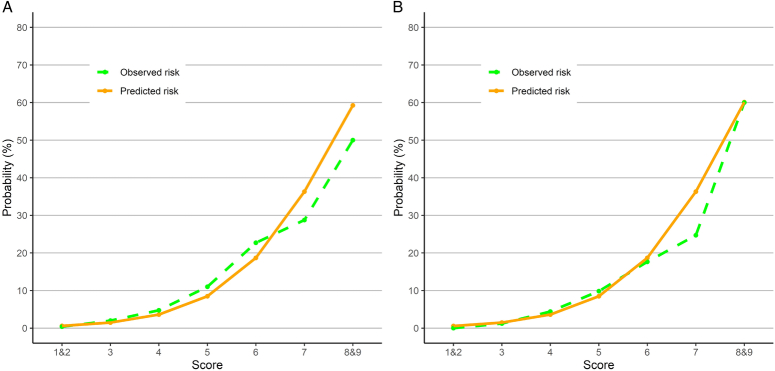
Calibration plots for the Simple PPUP score in the validation cohorts. The Simple PPUP score–based estimate of postoperative adverse events and the corresponding observed value for each point on the score (A for 2019 and B for 2020). Both the estimated and observed probability of postoperative adverse events gradually increased from almost 0% at a score of ≤2 to ~5% at a score of 4 and to >50% at a score of ≥8 in the two validation cohorts.

The discriminatory powers of the Simple PPUP score in the two validation cohorts were 0.812 (0.775–0.845) and 0.819 (0.782–0.852), respectively (Fig. S1, Supplemental Digital Content 4, http://links.lww.com/JS9/D289). The simple PPUP score–based estimates of postoperative adverse events and the corresponding observed values for each point on the score are shown in Figure 2. The estimated and observed probability of postoperative adverse events gradually increased from almost 0% at a score of ≤2 to >50% at a score of ≥8 in the two validation cohorts. Sensitivity analyses revealed that a higher Simple PPUP score was associated with various unfavorable outcomes, including less-severe adverse events (Table S4, Supplemental Digital Content 3, http://links.lww.com/JS9/D288).

A score of ≥5 exhibited a high NPV (97.5–97.9%) for postoperative adverse events in the two validation cohorts (Table 3 and Table S5, Supplemental Digital Content 3, http://links.lww.com/JS9/D288). Additionally, 1265 (69.2%) and 1127 (66.8%) patients in the two validation cohorts had a Simple PPUP score of ≤4, corresponding to a 3% risk of developing postoperative adverse events. Accordingly, a score of 5 was determined as a cutoff value for the Simple PPUP score.

**Table 3:** Cutoffs for simple PPUP score.

	Score								
	≥1	≥2	≥3	≥4	≥5	≥6	≥7	≥8	≥9
2019 cohort									
Sensitivity, %	100.0	100.0	99.3	90.6	76.8	53.6	26.8	10.1	0.0
Specificity, %	0.0	0.5	15.7	50.4	73.0	88.3	95.8	99.2	99.9
PPV, %	7.6	7.6	8.8	13.0	18.9	27.3	34.3	50.0	0.0
NPV, %	—	100.0	99.6	98.5	97.5	95.9	94.1	93.1	92.4
Case < threshold, *n*	0	8	266	865	1265	1556	1719	1799	1826
Postoperative adverse events < threshold, *n* (%)	0 (0.0)	0 (0.0)	1 (0.1)	13 (0.7)	32 (1.8)	64 (3.5)	101 (5.5)	124 (6.8)	138 (7.6)
2020 cohort									
Sensitivity, %	100.0	100.0	100.0	94.9	79.5	55.6	29.9	12.8	0.9
Specificity, %	0.0	1.1	13.9	45.3	70.2	86.6	95.5	99.4	99.9
PPV, %	6.9	7.0	8.0	11.4	16.6	23.6	33.0	60.0	50.0
NPV, %	—	100.0	100.0	99.2	97.9	96.3	94.8	93.9	93.1
Case < threshold, *n*	0	17	219	718	1127	1412	1582	1663	1686
Postoperative adverse events < threshold, *n* (%)	0 (0.0)	0 (0.0)	0 (0.0)	6 (0.4)	24 (1.4)	52 (3.1)	82 (4.9)	102 (6.0)	116 (6.9)

NPV, negative predictive value; PPV, positive predictive value.

The number of patients who underwent each type of surgery, including open laparotomy and laparoscopic surgery, were calculated with cutoff values for the Simple PPUP score (Table S6, Supplemental Digital Content 3, http://links.lww.com/JS9/D288). Approximately 17–19% of patients who had a Simple PPUP score of <5 underwent laparoscopic surgery in the two validation cohorts with minimal risks for postoperative adverse events.

## Discussion

This study developed a simple scoring system to predict postoperative severe adverse events of PPU with generalized peritonitis on a scale of 0–11, and a score of <5 predicted <3% probability of developing postoperative adverse events regardless of type of surgery. Therefore, the cutoff value of 5 would help surgeons to determine the type of surgery, which needs to be further validated. Notably, sensitivity analyses revealed that a higher Simple PPUP score was associated with other unfavorable outcomes, such as mortality and length of hospital stay. Hence, the system demonstrates high clinical utility as a disease severity scale.

Based on the derivation and validation processes, several beneficial features of the Simple PPUP score can be considered. First, the study utilized data from a nationwide database in Japan, which has the highest prevalence of peptic ulcer disease. The derivation cohort comprised over 8000 patients with PPU, encompassing more than 500 outcome events, whereas most previous studies on PPU scoring involved fewer than 300–500 patients^34–36^, indicating that the Simple PPUP score may offer better precision. Second, the primary outcome chosen was postoperative adverse events, which is more clinically relevant in recent years. According to a study on PULP score that involved patients with PPU requiring surgery, the mortality rate was 27% in 2009^37^. However, in the current study, the mortality rate was less than 8%, possibly reflecting improved surgical quality and critical care. The Simple PPUP score proves useful for evaluating surgical tolerance by assessing complications in addition to mortality. Third, the potential severity predictors were selected considering a core variable set generated by an international study group^5^. Although a few parameters could not be utilized owing to their unavailability in the NCD, this study analyzed globally accepted clinical factors for PPU.

Moreover, the simple PPUP score demonstrated high clinical feasibility. The system, requiring only preoperative information, enables risk stratification for patients with PPU before surgery. While the current study cannot determine the superiority of type of surgery, >1 out of 5–6 patients (>17–19%) underwent laparoscopic surgery with a minimal likelihood of postoperative complications when a score was <5, which would probably aid surgeons in determining appropriate treatment strategies. Notably, >60% of patients can be categorized into this low-risk category. Furthermore, unlike some preexisting mortality prediction models that include time to surgery^32^, the simple PPUP score does not incorporate variables subject to change before surgery, such as vital signs and time to surgery. All six factors in the Simple PPUP score can be assessed preoperatively and consistently until surgery, with sufficient discrimination and calibration even without time-dependent variables.

Notably, the simple PPUP score does not negate the necessity to reevaluate the disease severity of patients based on intraoperative findings. Given that the simple PPUP score was developed using retrospective data and tailored management, it should only be used to predict outcomes before surgery, and postoperative treatments should not be modified based on the score. Additionally, although a score <5 may be practical for selecting the type of surgery, an optimal surgical strategy depending on the Simple PPUP score should be confirmed in another study.

This study had several limitations that should be considered when interpreting the findings. The study utilized retrospective data from the NCD, which lacked records on the indications for emergency surgery for PPU and selection/information bias should be cautioned. Although generalized peritonitis is a validated indication for emergency surgery, our results may differ if unrecorded/missing factors, such as the availability of operating rooms or surgeons, influence the reasons for emergency surgery. Additionally, clinical information during surgery and postoperative management was not available. Although most potential severity predictors were assessed during the development of the score in this study, differences in postoperative treatment between surgeons and institutions can impact its predictive ability. Furthermore, the reliability of the simple PPUP score is significantly dependent on the quality of the NCD data. Although annual audits maintain data reliability to some extent^19^, generalizability should be confirmed through external validation using a different dataset. Finally, as the discriminatory power (C-statistics) of the Simple PPUP score is only approximately 0.8, similar to other reported scoring systems^32^, its clinical utility should be cautiously interpreted. Middle-range scores (5 or 6) could neither definitively determine nor rule out the risk of postoperative adverse events.

In conclusion, this study reported the derivation and internal validation of the simple PPUP score, a severity score for PPU with generalized peritonitis. The tool comprises six clinical parameters: age, dyspnea at rest, preoperative sepsis, class III/IV/V based on the ASA-PS classification system, and albumin and creatinine levels, with scores ranging from 0 to 11. Patients with a simple PPUP score of <5 have a <3% probability of developing postoperative adverse events and would be clinically useful. A comparison of surgical strategies based on the simple PPUP score, as well as the validation of the generalizability of the score, would be required in future studies.

## Ethical approval

All collaborating hospitals obtained individual local institutional review board approval for patient enrollment in the NCD. The current study was approved by the Ethics Committee for Conduct of Human Research of Keio University School of Medicine (application number: 20211020) before study initiation.

## Consent

The need for informed consent was waived due to data anonymity.

## Source of funding

There is no funding on this study.

## Author contribution

R.Y., T.M., H.K., and Y.K.: conceptualized the study; R.Y., S.H., H.T., H.K., and H.H.: designed the study; K.S. and Y.K.: performed data quality management and data curation; Y.K. and J.S.: managed quality control of the study; S.H. and H.T.: performed data analysis; R.Y. and S.H.: conducted interpretation, writing, and critical revision. All authors revised the manuscript.

## Conflicts of interest disclosure

Shinya Hirakawa belongs to an endowed course funded by Takeda Pharmaceutical Company, Limited and to a department that accepts financial support from the National Clinical Database, Johnson & Johnson K.K., Nipro Corporation, and Intuitive Surgical Sàrl. Hisateru Tachimori belongs to an endowed course funded by Takeda Pharmaceutical Company, Limited and to a department that accepts financial support from the National Clinical Database, Johnson & Johnson K.K., Nipro Corporation, and Intuitive Surgical Sàrl. All JTACS Disclosure forms have been supplied and are provided as supplemental digital content.

## Research registration unique identifying number (UIN)

The study was retrospectively registered at the University Hospital Medical Information Network Clinical Trial Registry (UMIN000053040).

## Guarantor

Ryo Yamamoto.

## Data availability statement

Data on individual surgical cases in this study are not publicly available. Aggregate data, including data reported in this study, can be accessed by submitting a research plan to the National Clinical Database (NCD) Office and requesting access, commonly via an NCD-related society (such as the Japanese Society of Gastroenterological Surgery). If the proposal is approved, the deidentified data (including participants and related data, if necessary) can be assessed by a statistical specialist affiliated with the NCD.

## Provenance and peer review

Not invited.

## Supplementary Material
