# Supplementary material for: Simple severity scale for perforated peptic ulcer with generalized peritonitis: a derivation and internal validation study
Source: Int J Surg. 2024 Aug 8;110(11):7134–41. doi: 10.1097/JS9.0000000000002037 (PMC11573046; doi:10.1097/JS9.0000000000002037)
Supplement: Supplementary file 2 [file js9-110-7134-s002.docx]

**Title: Simple Severity Scale for Perforated Peptic Ulcer with Generalized Peritonitis: A Derivation and Internal Validation Study**

**Authors: Ryo Yamamoto, et al.**

**Supplementary Data S1: Categorization of the variables**

**Categorization of variables using standard values**

For optimal categorization, single-variable logistic regression for the primary outcome was performed using categorized numerical values.

- For the criteria (thresholds) for categorization, a restricted cubic spline (RCS) function of variables was used in the single-variable logistic regression for the primary outcome.

Categorization was determined by observing the categorization plots, and the following steps were taken:

1. Preparation of data
   - Use training data (2013–2018 data).
   - Missing, unknown, and unrecorded values were excluded.
   - The observed probability was applied to the standard value.
   - Abnormal values were classified as “less than the standard value” and “greater than the standard value.”
   - Winsorizing was performed on the values: A value of 1%tile or less for “less than the standard value” was assumed to be a value of 1%tile, and a value of 99%tile or more for “greater than the standard value” was assumed to be a value of 99%tile.
   - If the number of outcome/nonoutcome events for “less than the standard value” or “greater than the standard value” is less than 5, then it is combined with the “standard value.”
2. A univariate logistic regression for the outcome using the RCS of variables was performed to obtain the log-likelihood ratio of the constant model.
   - Use the RCS from the rms package in R.
   - The number of knots was set to 5. The default knot positions were 5%, 27.5%, 50%, 72.5%, and 95%.
   - If an error occurs, use the RCS for the square root, following Harrell’s comments.
3. Univariate logistic regression for the outcome using categorized variables was performed to obtain the log-likelihood ratio of the constant model.
   - Categorization was based on the categorization in previous studies using NCD; the categorization of the “less than standard, standard, and greater than standard” levels that are predefined in CRF in accordance with the general population, and the categorization of previous non-NCD studies.
4. The results of step 2 (RCS) were compared with those of step 3, and optimal thresholds were selected for categorization.
   - The RCS plots were compared with the plots for each categorization; RCS could overfit the data because of its flexibility.
   - The log-likelihood ratio of the RCS (step 2) was compared with the log-likelihood ratio of each categorization (step 3); a categorization close to the log-likelihood ratio of the RCS was considered a well-fitting transformation.
   - Consistency of the categorization with clinical knowledge was also examined.

Each variable was categorized as follows:

1. Hemoglobin, male
   - Hemoglobin in males was categorized as less than standard, standard, and greater than standard.
     - Categorization was performed using the CRF reference values.
   - The reference value for males is in the range of 13.5–17 g/dL.
   - The vertical red lines represent the upper and lower limits of the standard values.
   - Blue dots represent the RCS values.


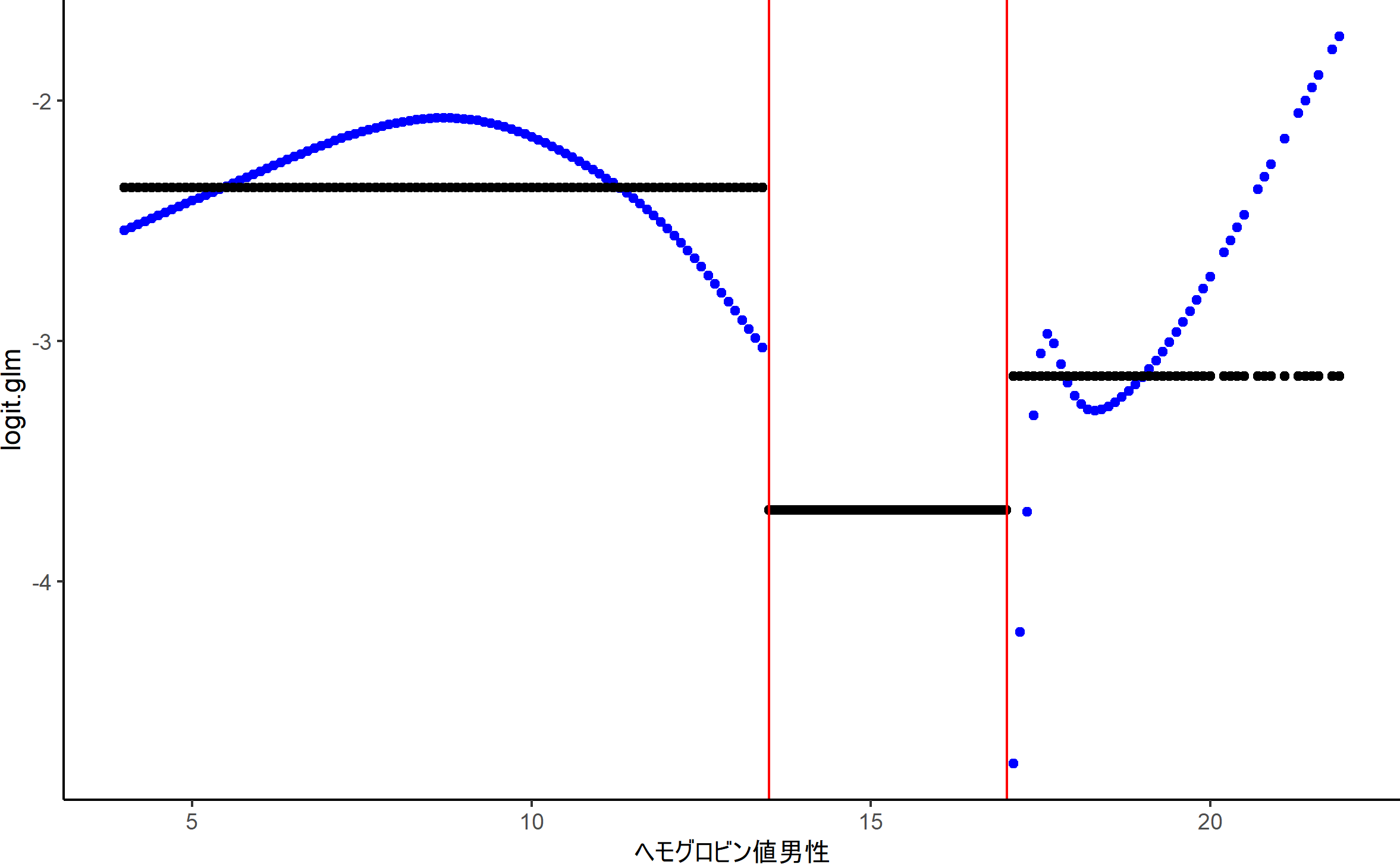


Hemoglobin (g/dL)

1. Hemoglobin female
   - Hemoglobin in female was categorized as less than standard, standard, and greater than standard.
     - Categorization was performed using the CRF reference values.
   - The reference value for women is in the range of 11.5–15 g/dL.
   - The vertical red lines represent the upper and lower limits of the standard values.
   - The blue dots represent the RCS values.


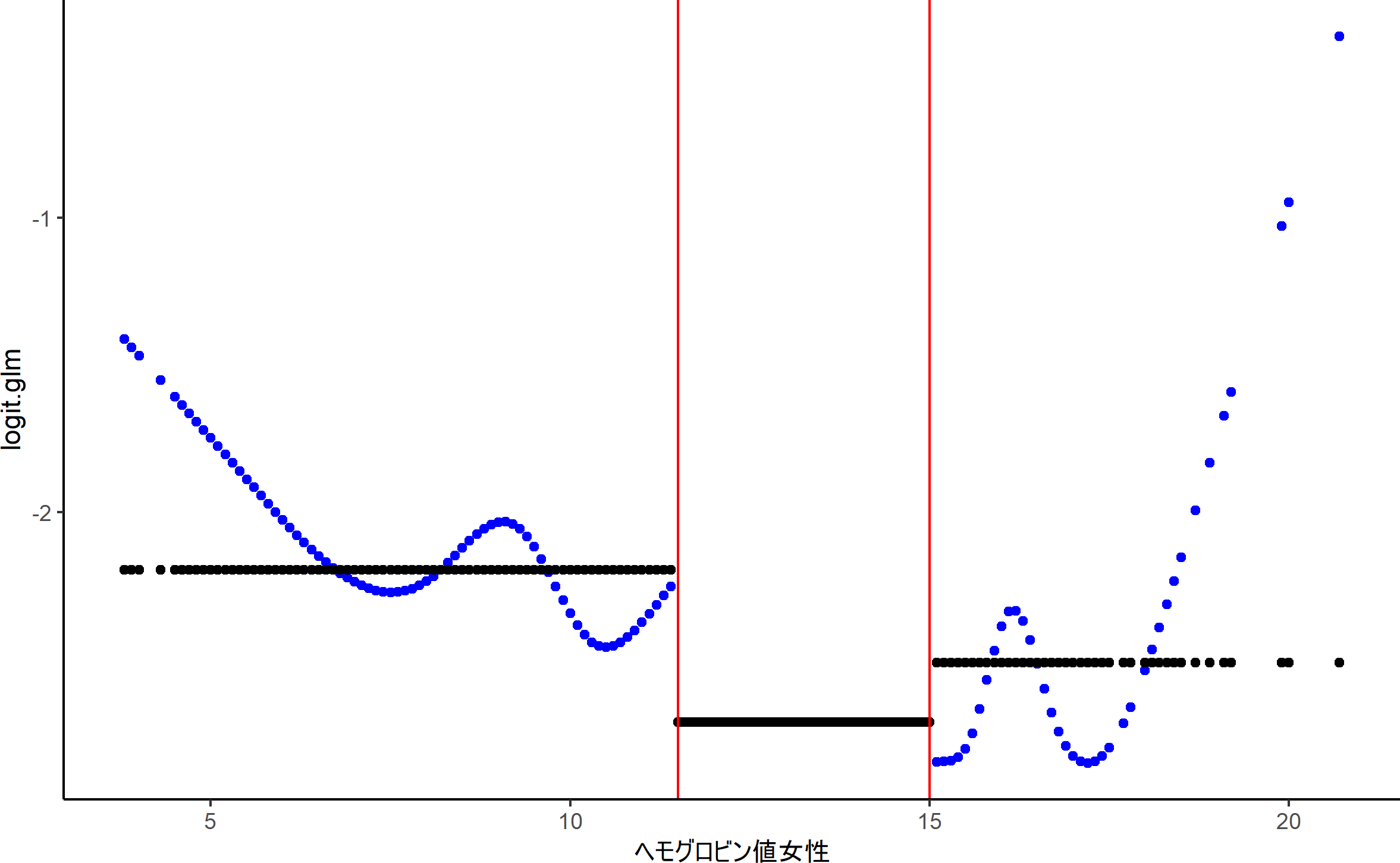


Hemoglobin (g/dL)

1. Albumin
   - Albumin was categorized into Alb < 2.0, 2.0 ≤ Alb < 3.0, and 3.0 ≤ Alb.
     - Categorization was performed on the basis of previous studies using NCD.
   - The standard value was in the range of 4.0–5.0 g/dL, which belonged to 3.0 ≤ Alb.
   - Considering that the number of “greater than the standard value” events was less than 5, it is merged with the “standard value.”
   - The vertical red lines represent the upper and lower limits of the standard value.
   - The blue dots represent the RCS values.


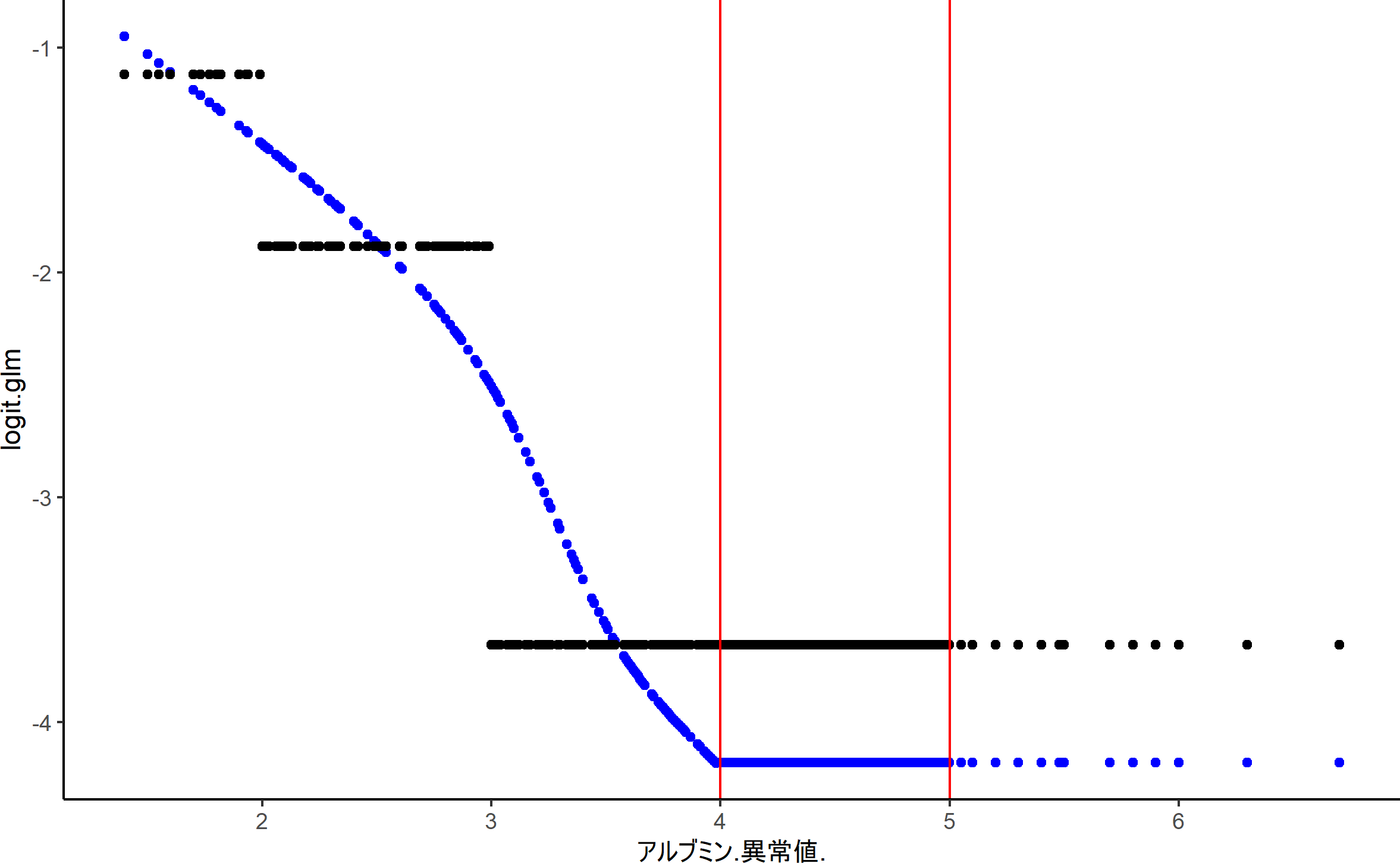


Albumin (g/dL)

1. Urea nitrogen
   - Urea nitrogen was categorized into BUN ≤ 20 and 20 < BUN.
     - Categorization by the CRF reference value was performed.
   - The standard value was in the range of 8–20 mg/dL, which belonged to BUN ≤ 20.
   - Considering that the number of events in the “less than standard value” category was less than 5, it was merged with the “standard value” category.
   - The vertical red lines represent the upper and lower limits of the standard value.
   - The blue dots represent the RCS values.


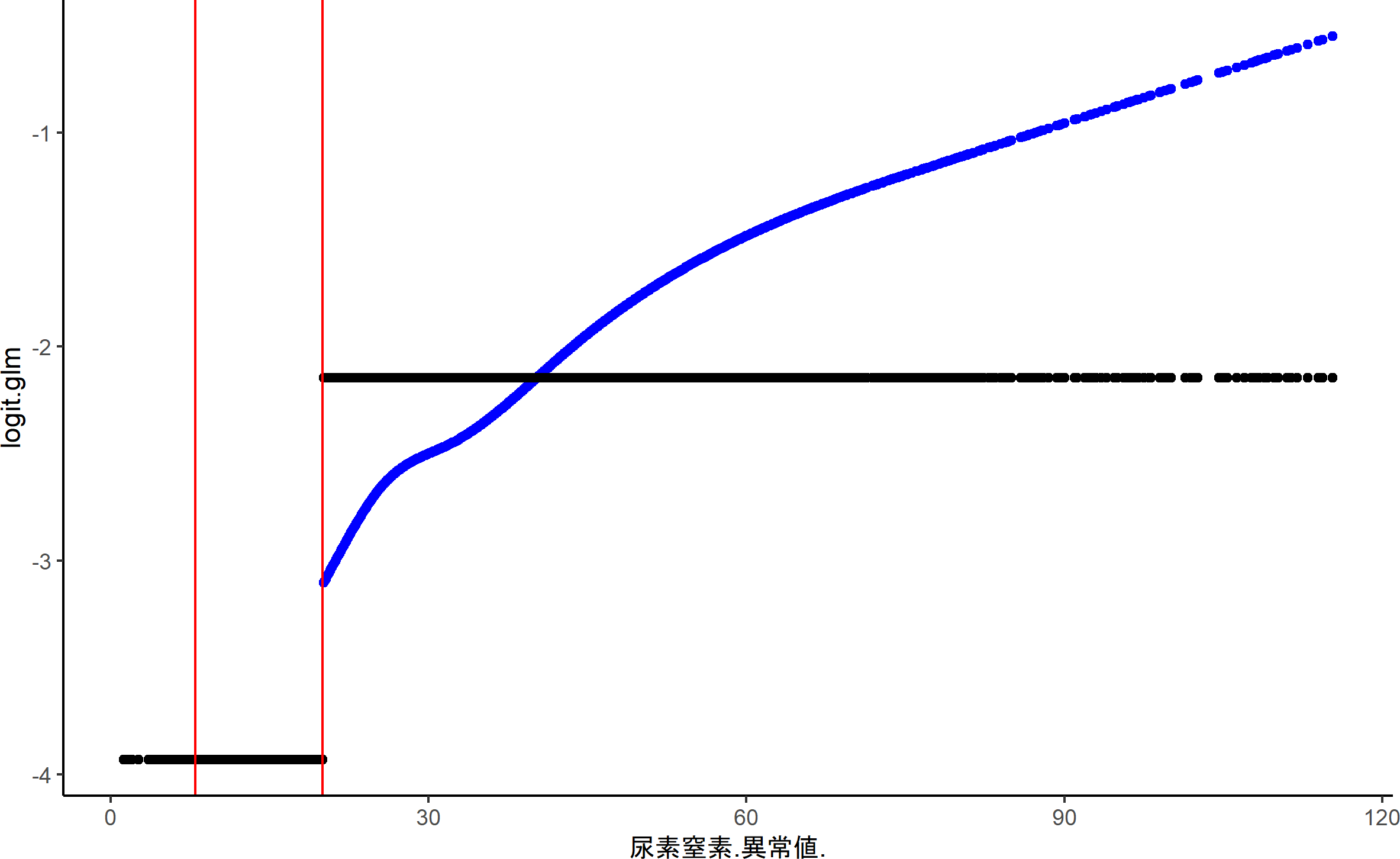


Urea nitrogen (mg/dL)

1. CRP
   - CRP was categorized as CRP ≤ 1.0 and 1.0 < CRP.
     - The Glasgow prognostic score was used as a reference for categorization.
   - The standard value was less than 0.1 mg/dL, which belonged to CRP ≤ 1.0.
   - The vertical red lines represent the upper and lower limits of the standard values.
   - The blue dots represent the RCS values. The RCS of the square root was used.


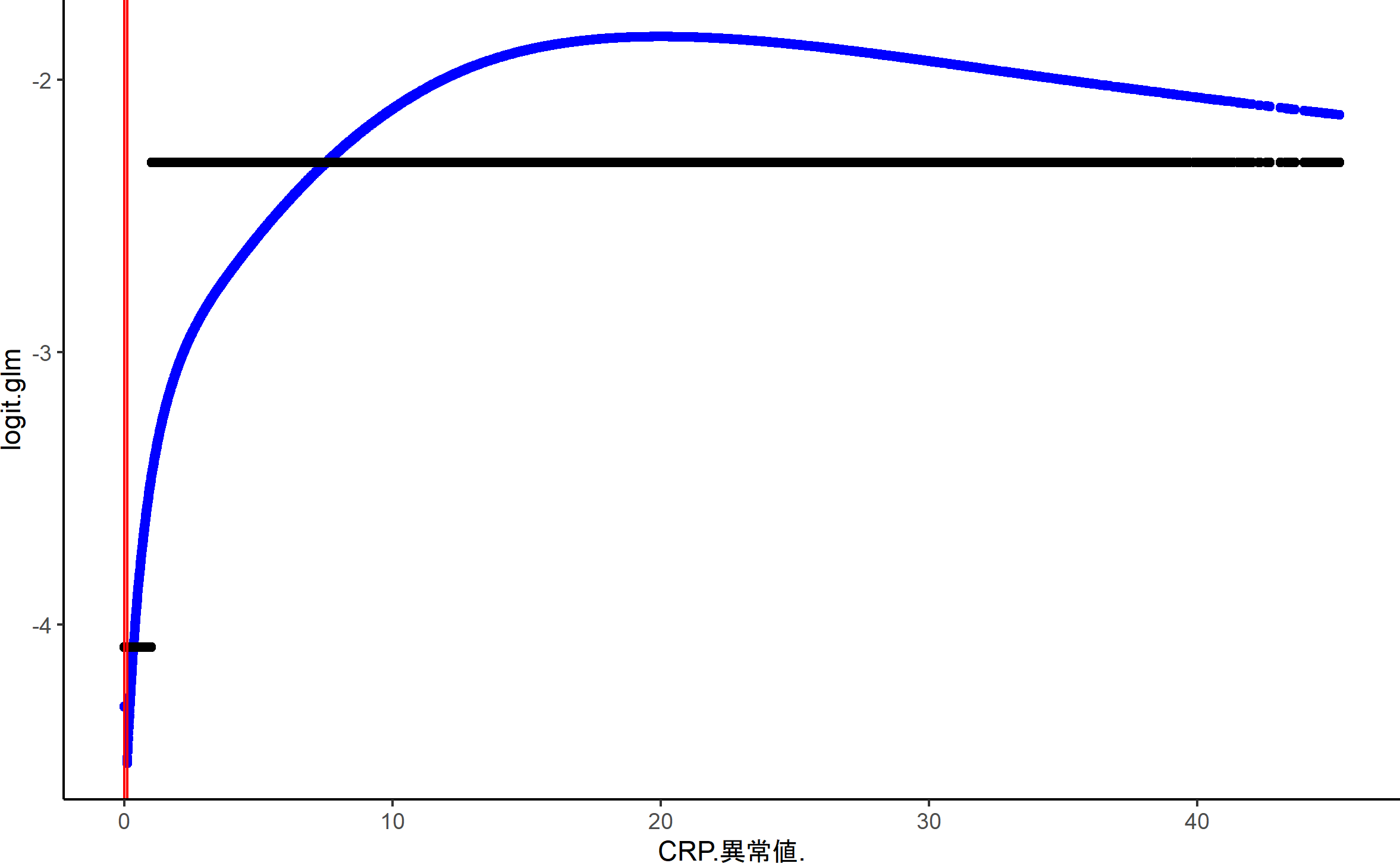


CRP (mg/dL)

**Transformation of variables without standard values**

Single-variable logistic regressions for the outcome using transformed variables (creatinine, age at surgery, and BMI) were performed, and the optimal transformation was selected.

- As a criterion for variable transformation, the RCS function of variables was used in the single-variable logistic regression for the primary outcome.

Categorization or variable transformation was determined by observing the variable transformation plots.

1. Preparation of data to be used.
   - Training data from 2013 to 2018 was used.
   - Missing, unknown, and unrecorded values were excluded.
   - Winsorizing was performed on the values: A value of 1%tile or less was assumed to be a value of 1%tile, and a value of 99%tile or more was assumed to be a value of 99%tile.
2. A univariate logistic regression for the outcome was performed using the RCS of variables to obtain the log-likelihood ratio to the constant model.
   - RCS from the rms package in R was used.
   - The number of knots was set to 5. The default knot positions were 5%, 27.5%, 50%, 72.5%, and 95%.
   - If an error occured, RCS for the square root was used, following Harrell’s comments.
3. A univariate logistic regression for the outcome using the transformed variables (untransformed, logarithmic, square root, squared, and inverse [with categorization for BMI]) was performed to obtain the log-likelihood ratio to the constant model.
   - The categorization was based on the categorization in previous studies using NCD and the categorization of previous non-NCD studies.
4. Compare the results of step 2 (RCS) with those of step 3, and select the optimal transformation of variables.
   - The RCS plots were compared with the plots for each transformed variable; RCS was considered to overfit the data because of its flexibility.
   - The log-likelihood ratio of the RCS (step 2) was compared with the log-likelihood ratio of each method for transformation (step 3); transformation close to the log-likelihood ratio of the RCS was considered a well-fitting transformation.
   - If no significant difference in the log-likelihood ratio was found, then simplest possible transformation was chosen. Preference was given in the following order: no transform > logarithm greater than square root > square > inverse.
   - The consistency of the variable transformation with clinical knowledge was also examined.

Each variable was transformed as follows:

1. Creatinine
   - Logarithmic transformation was performed.
   - The blue dots represent the RCS values. The RCS of the square root was used.


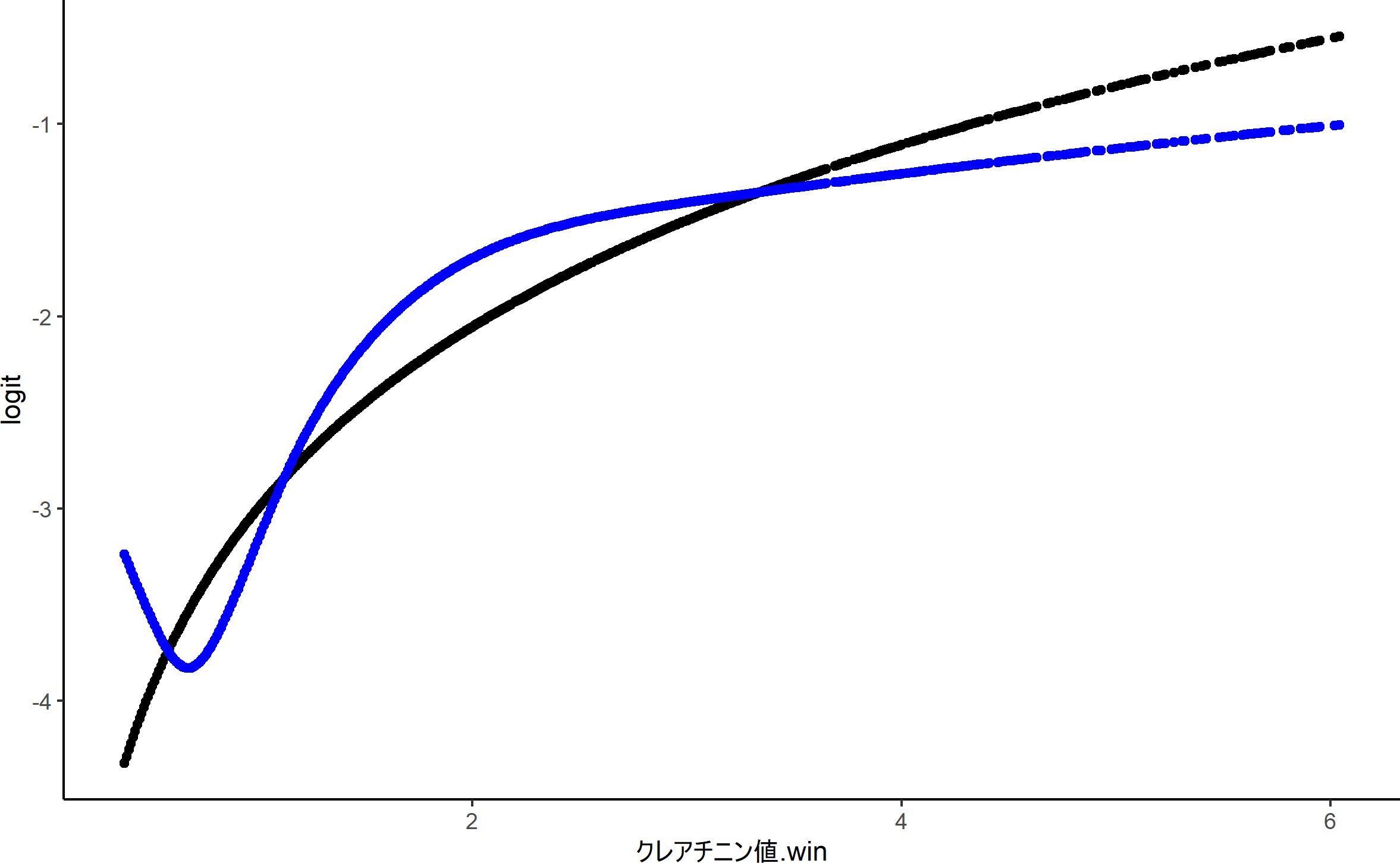


Creatinine (mg/dL)

1. Age at the time of surgery
   - The data were used in the analysis without variable conversion. However, considering that the change per year was small, the value divided by 10 was used in the analysis.
   - The blue dots represent the RCS values.


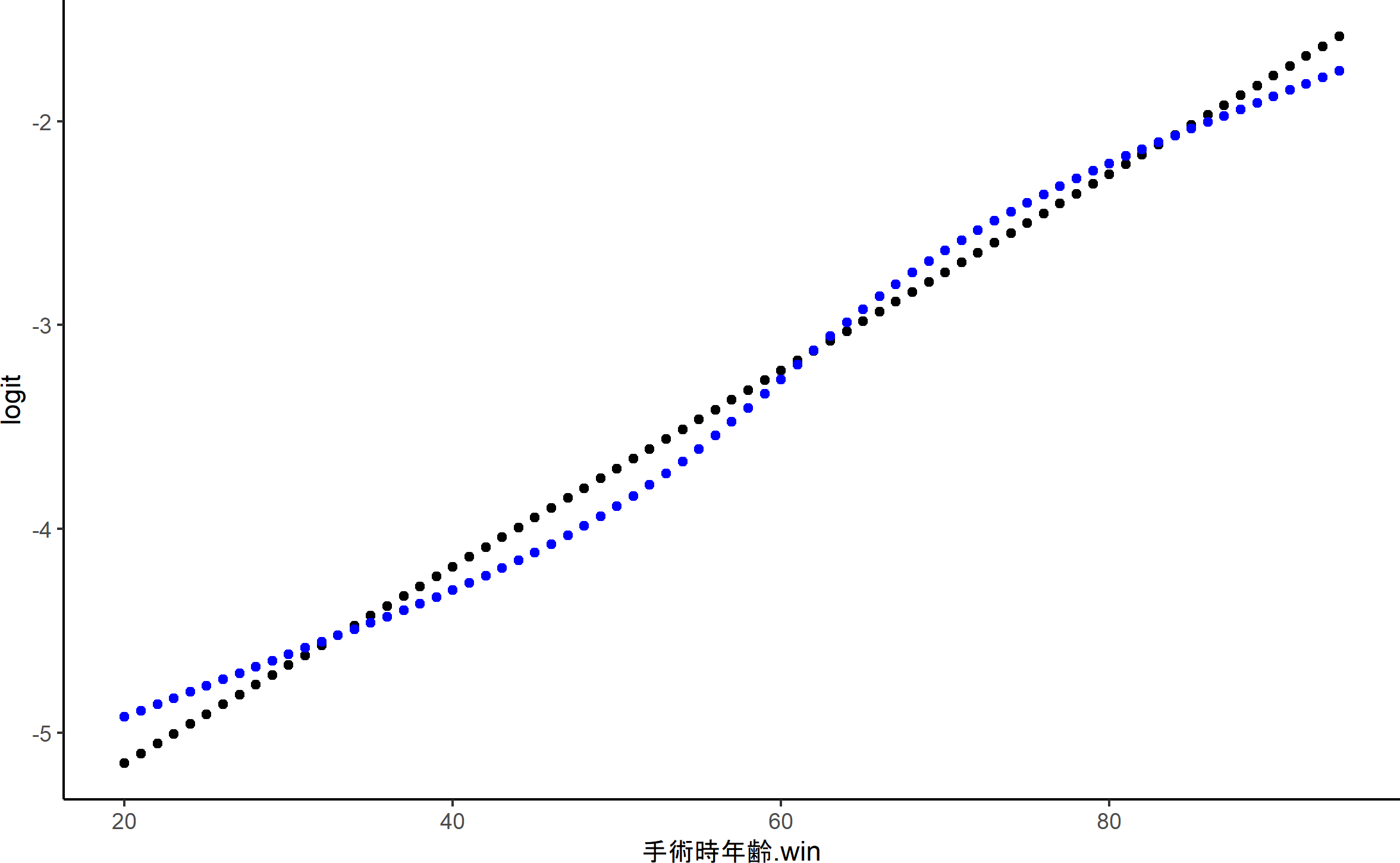


Age (years)

1. BMI
   - BMI was categorized into BMI < 18.5, 18.5 ≤ BMI < 25, and 25 ≤ BMI.
     - Categorization was performed with reference to the criteria suggested by the Japan Society for the Study of Obesity.
   - The blue dots represent the RCS values.


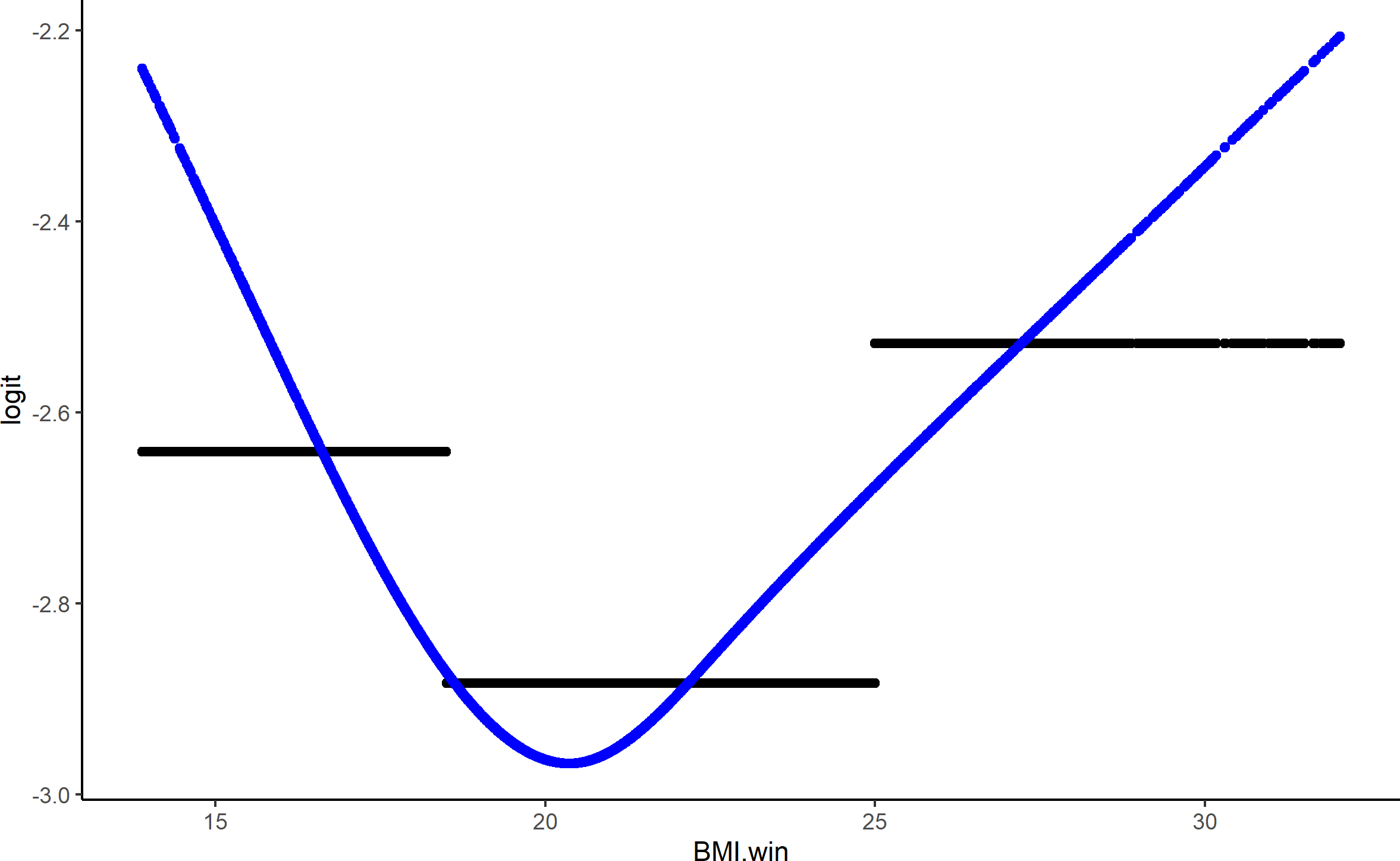


BMI (kg/m^2^)
