# Supplementary material for: Simple severity scale for perforated peptic ulcer with generalized peritonitis: a derivation and internal validation study
Source: Int J Surg. 2024 Aug 8;110(11):7134–41. doi: 10.1097/JS9.0000000000002037 (PMC11573046; doi:10.1097/JS9.0000000000002037)
Supplement: Supplementary file 3 [file js9-110-7134-s003.docx]

| Table S1. Core variables for perforated peptic ulcer | | |
| --- | --- | --- |
| Core descriptor set* | Availability in NCD | Surrogate variable |
| **Demographic characteristics and comorbidities** |  |  |
| Presence of chronic comorbidities | Yes |  |
| Age | Yes |  |
| Use of anticoagulants | Yes |  |
| Presence of immunosuppression | Yes |  |
| **Not clustered risk factors for PPU** |  |  |
| Previous gastric surgery | No | No |
| Diagnosis of upper gastrointestinal cancer | Excluded from study |  |
| Use of ulcerogenic drugs | Partially yes |  |
| Nonsteroidal anti-inflammatory drug use | No | No |
| Steroid use | Yes |  |
| Previous peptic ulcer disease | No | No |
| **Presentation and pathway factors** |  |  |
| Time from symptom to admission | No |  |
| Time from admission to theaters | Yes |  |
| Systolic blood pressure on admission | No | ASA-PS |
| Heart rate on admission | No | ASA-PS |
| **Need for organ support** |  |  |
| Presence of organ failure | Yes |  |
| HDU support requirement on admission | Partially yes |  |
| Invasive ventilation requirement on admission | No | ASA-PS |
| **Biochemical parameters** |  |  |
| Score on intensive care mortality predictor tool | No | No |
| Lactate level on admission | Partially yes |  |
| **Prognostic tools** |  |  |
| Score on postoperative mortality predictor tool | Yes |  |
| Score on a measure of frailty tool | Partially yes |  |
| Summary score of comorbidities | Partially yes |  |
| ASA fitness grade | Yes |  |
| **Perforation details** |  |  |
| Presence of generalized peritonitis | Not used intentionally |  |
| Type of peritoneal contamination at operation | Not used intentionally |  |
| Site of perforation | Not used intentionally |  |
| Size of defect | Not used intentionally |  |
| NCD, National Clinical Database; PPU, perforated peptic ulcer; HDU, high-dependency unit. *PPU CDS collaborators generated a core descriptor set for perforated peptic ulcer by the Delphi method in 2022. | | |
|  |  |  |
|  |  |  |

| Table S2. Inclusion frequency of variables in the simple BLaF model | | |
| --- | --- | --- |
| Variables | Inclusion frequency | Selected in the final model* |
| Preoperative sepsis | 2000 | Yes |
| ASA-PS, class III/IV/V | 2000 | Yes |
| Albumin level, 2.0–3.0 g/dL | 2000 | Yes |
| Albumin level, < 2.0 g/dL | 2000 | Yes |
| Creatinine level, mg/dL, log | 2000 | Yes |
| Respiratory distress - dyspnea at rest | 1995 | Yes |
| Age, years, 10^−1^ | 1992 | Yes |
| Congestive heart failure | 1686 | No |
| Acute kidney injury | 1608 | No |
| BUN level, > 20 mg/dL | 1585 | No |
| Dependent ADL | 1224 | No |
| Long-term steroid use | 595 | No |
| CRP, > 1.0 mg/dL | 579 | No |
| Metastatic carcinoma | 449 | No |
| Anticoagulants | 204 | No |
| Cerebrovascular disease | 70 | No |
| Hemoglobin, less than the normal range** | 67 | No |
| COPD | 59 | No |
| Hemodialysis | 22 | No |
| Preoperative chemotherapy within 90 days | 8 | No |
| Preoperative immunotherapy within 30 days | 8 | No |
| Male sex | 4 | No |
| Respiratory distress - dyspnea on exertion | 1 | No |
| Post-PCI | 1 | No |
| Emergency transport | 0 | No |
| Emergency surgery | 0 | No |
| Diabetes | 0 | No |
| Habitual drinking | 0 | No |
| BMI, 18.5–25 kg/m^2^ | 0 | No |
| BMI, ≥ 25 kg/m^2^ | 0 | No |
| Hemoglobin, greater than normal range** | 0 | No |
| BLaF, bootstrap resampling- Lasso selection on bootstrap data and fit the final model based on variables with inclusion frequency; ASA-PS, American Society of Anesthesiologists physical status; BUN, blood urea nitrogen; ADL, activity of daily living; CRP, C-reactive protein; COPD, chronic obstructive pulmonary disease; PCI, percutaneous coronary intervention; and BMI, body mass index. *Variables with ≥90% of inclusion frequency (≥1800 times) were selected into the final model. **Normal ranges of hemoglobin level are 13.5–17 g/dL in men and 11.5–15 in women. | | |
|  |  |  |
|  |  |  |
|  |  |  |
|  |  |  |
|  |  |  |

| Table S3. Variables in the final model | | | | |
| --- | --- | --- | --- | --- |
|  | Coefficient | Odds ratio | 95% CI | P value |
| Age, years, 10^−1^ | 0.28 | 1.32 | 1.22–1.42 | < 0.001 |
| Respiratory distress - none | Reference |  |  |  |
| Respiratory distress - dyspnea on exertion | −0.06 | 0.94 | 0.56–1.59 | 0.822 |
| Respiratory distress - dyspnea at rest | 1.11 | 3.02 | 2.08–4.38 | < 0.001 |
| Preoperative sepsis - no | Reference |  |  |  |
| Preoperative sepsis - yes | 0.82 | 2.26 | 1.77–2.90 | < 0.001 |
| ASA-PS, class I/II | Reference |  |  |  |
| ASA-PS, class III/IV/V | 1.19 | 3.27 | 2.56–4.18 | < 0.001 |
| Albumin level, ≥ 3.0 g/dL | Reference |  |  |  |
| Albumin level, 2.0–3.0 g/dL | 0.95 | 2.59 | 2.07–3.25 | < 0.001 |
| Albumin level, < 2.0 g/dL | 1.65 | 5.22 | 3.79–7.18 | < 0.001 |
| Creatinine level, mg/dL, log | 0.70 | 2.01 | 1.74–2.31 | < 0.001 |
| CI, confidence interval; ASA-PS, American Society of Anesthesiologists physical status. | | | | |
|  |  |  |  |  |

| Table S4. Association between Simple PPUP and outcomes | | | | |
| --- | --- | --- | --- | --- |
|  | Validation with the 2019 cohort | | Validation with the 2020 cohort | |
|  | OR/coefficient | 95% CI | OR/coefficient | 95% CI |
| Postoperative adverse event* | 2.19 | 1.93–2.48 | 2.30 | 2.00–2.64 |
| Length of hospital stay, days** | 6 | 6–7 | 8 | 7–8 |
| Prolonged hospital stay*** | 2.03 | 1.84–2.24 | 2.25 | 2.03–2.50 |
| 30-Day mortality | 2.21 | 1.91–2.56 | 2.56 | 2.17–3.02 |
| In-hospital mortality | 2.38 | 2.09–2.72 | 2.39 | 2.07–2.74 |
| OR, odds ratio; CI, confidence interval. *Postoperative severe adverse events defined as IV or V based on the Clavien–Dindo classification system. ** Only the length of hospital stay among survivors was calculated. ***Prolonged hospital stay was defined as stay for >30 days, and it was only assessed among survivors. | | | | |
|  |  |  |  |  |
|  |  |  |  |  |

| Table S5. Cutoffs for Simple PPUP score to predict grade III/VI/V adverse events of the Clavien–Dindo classification | | | | | | | | | |
| --- | --- | --- | --- | --- | --- | --- | --- | --- | --- |
|  | Score | | | | | | | | |
|  | ≥ 1 | ≥ 2 | ≥ 3 | ≥ 4 | ≥ 5 | ≥ 6 | ≥ 7 | ≥ 8 | ≥ 9 |
| **2019 cohort** |  |  |  |  |  |  |  |  |  |
| Sensitivity, % | 100.0 | 100.0 | 99.0 | 83.6 | 64.1 | 41.9 | 19.5 | 6.4 | 0.0 |
| Specificity, % | 0.0 | 0.5 | 17.2 | 53.4 | 75.7 | 90.5 | 96.7 | 99.4 | 99.9 |
| PPV, % | 16.3 | 16.4 | 18.9 | 25.9 | 34.0 | 46.1 | 53.7 | 67.9 | 0.0 |
| NPV, % | - | 100.0 | 98.9 | 94.3 | 91.5 | 88.9 | 86.0 | 84.5 | 83.7 |
| Case < threshold, n | 0 | 8 | 266 | 865 | 1265 | 1556 | 1719 | 1799 | 1826 |
| Postoperative adverse events < threshold, n (%) | 0 (0.0) | 0 (0.0) | 3 (0.2) | 49 (2.7) | 107 (5.9) | 173 (9.5) | 240 (13.1) | 279 (15.3) | 298 (16.3) |
| **2020 cohort** |  |  |  |  |  |  |  |  |  |
| Sensitivity, % | 100.0 | 100.0 | 98.4 | 84.8 | 65.7 | 38.5 | 18.4 | 5.8 | 0.3 |
| Specificity, % | 0.0 | 1.2 | 15.5 | 48.7 | 74.0 | 88.6 | 96.4 | 99.5 | 99.9 |
| PPV, % | 18.3 | 18.5 | 20.7 | 27.0 | 36.2 | 43.1 | 53.8 | 72.0 | 50.0 |
| NPV, % | - | 100.0 | 97.7 | 93.5 | 90.6 | 86.5 | 84.1 | 82.5 | 81.7 |
| Case < threshold, n | 0 | 17 | 219 | 718 | 1127 | 1412 | 1582 | 1663 | 1686 |
| Postoperative adverse events < threshold, n (%) | 0 (0.0) | 0 (0.0) | 5 (0.3) | 47 (2.8) | 106 (6.3) | 190 (11.3) | 252 (14.9) | 291 (17.2) | 308 (18.2) |
| PPV, positive predictive value; NPV, negative predictive value | | | | | | | | | |
|  |  |  |  |  |  |  |  |  |  |

| Table S6. Cutoffs for Simple PPUP score and surgical procedures | | | | | | | | |
| --- | --- | --- | --- | --- | --- | --- | --- | --- |
|  | Score | | | | | | | |
|  | ≤ 1 | ≤ 2 | ≤ 3 | ≤ 4 | ≤ 5 | ≤ 6 | ≤ 7 | ≤ 8 |
| **2019 cohort** |  |  |  |  |  |  |  |  |
| Open laparotomy, n (%) | 4 (66.7) | 205 (77.1) | 657 (80.1) | 962 (82.3) | 1185 (83.4) | 1310 (83.9) | 1378 (84.4) | 1397 (84.5) |
| Omental patch | 3 | 172 | 522 | 745 | 908 | 992 | 1031 | 1044 |
| Primary closure | 1 | 30 | 121 | 189 | 236 | 268 | 293 | 297 |
| Partial gastrectomy | 0 | 3 | 12 | 26 | 38 | 46 | 49 | 51 |
| Total gastrectomy | 0 | 0 | 2 | 2 | 3 | 4 | 5 | 5 |
| Laparoscopic surgery, n (%) | 2 (33.3%) | 61 (22.9) | 163 (19.9) | 207 (17.7) | 236 (16.6) | 252 (16.1) | 255 (15.6) | 256 (15.5) |
| Omental patch/primary closure | 2 | 61 | 162 | 206 | 235 | 251 | 254 | 255 |
| Partial gastrectomy | 0 | 0 | 1 | 1 | 1 | 1 | 1 | 1 |
| **2020 cohort** |  |  |  |  |  |  |  |  |
| Open laparotomy, n (%) | 12 (63.2) | 172 (78.9) | 546 (78.8) | 853 (81.4) | 1073 (82.7) | 1195 (83.2) | 1262 (83.6) | 1282 (83.7) |
| Omental patch | 9 | 144 | 427 | 649 | 804 | 883 | 928 | 941 |
| Primary closure | 3 | 26 | 109 | 190 | 244 | 282 | 301 | 307 |
| Partial gastrectomy | 0 | 2 | 10 | 13 | 24 | 29 | 32 | 33 |
| Total gastrectomy | 0 | 0 | 0 | 1 | 1 | 1 | 1 | 1 |
| Laparoscopic surgery, n (%) | 7 (36.8) | 46 (21.1) | 147 (21.2) | 195 (18.6) | 224 (17.3) | 242 (16.8) | 247 (16.4) | 249 (16.3) |
| Omental patch/primary closure | 7 | 46 | 147 | 195 | 224 | 242 | 247 | 249 |
| Partial gastrectomy | 0 | 0 | 0 | 0 | 0 | 0 | 0 | 0 |
| PPV, positive predictive value; NPV, negative predictive value | | | | | | | | |
|  |  |  |  |  |  |  |  |  |
