# Supplementary material for: Simple severity scale for perforated peptic ulcer with generalized peritonitis: a derivation and internal validation study
Source: Int J Surg. 2024 Aug 8;110(11):7134–41. doi: 10.1097/JS9.0000000000002037 (PMC11573046; doi:10.1097/JS9.0000000000002037)
Supplement: Supplementary file 4 [file js9-110-7134-s004.docx]

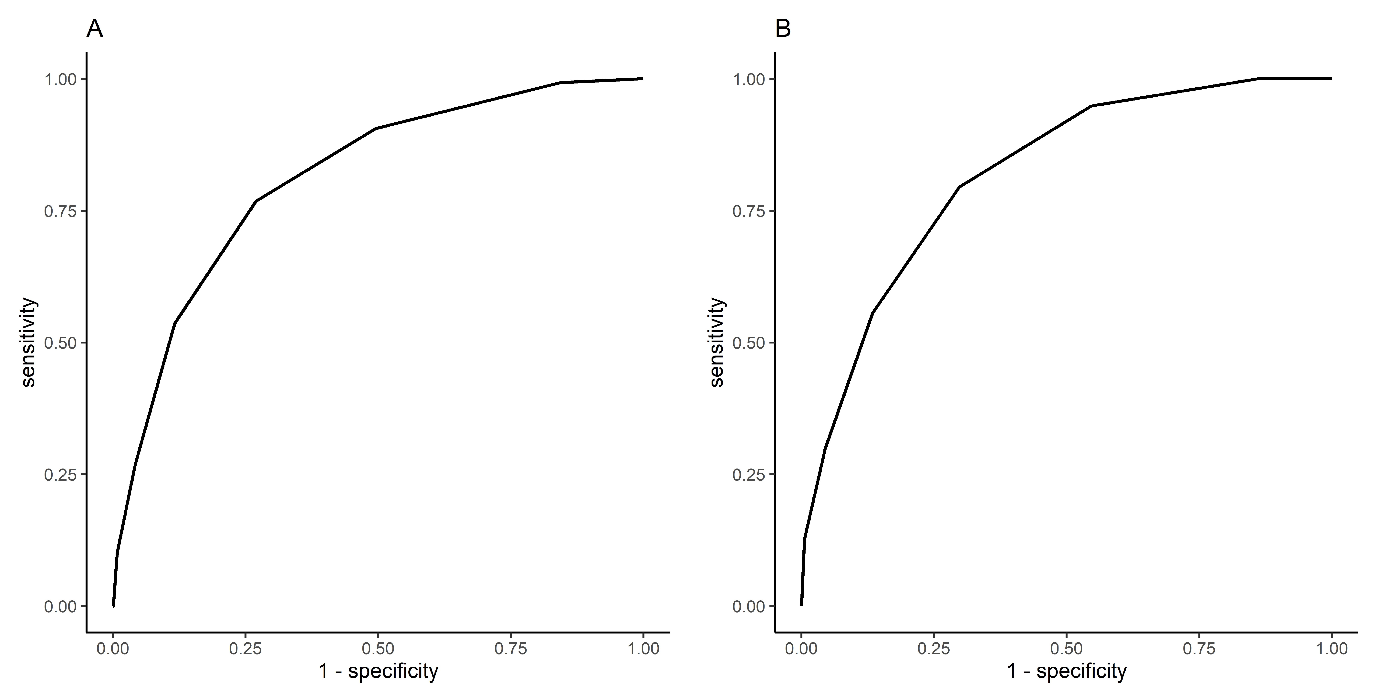


**Figure S1. Receiver operating characteristic curve for the Simple PPUP score in the validation cohorts**

Receiver operating characteristic curves for the Simple PPUP score in the 2019 (A) and 2020 (B) validation cohorts. The discriminatory powers (c-statistics) in the two validation cohorts were 0.812 (0.775–0.845) and 0.819 (0.782–0.852), respectively.
